# Butyrate Selectively Targets Super‐Enhancers and Transcriptional Networks Associated with Human Mast Cell Function

**DOI:** 10.1002/eji.202451680

**Published:** 2025-06-11

**Authors:** Jelle Folkerts, Marjolein J. W. de Bruijn, Wilfred F. J. van IJcken, Rudi W. Hendriks, Ralph Stadhouders

**Affiliations:** ^1^ Department of Pulmonary Medicine Erasmus MC Erasmus University Medical Center Rotterdam Rotterdam The Netherlands; ^2^ Department of Pathology Stanford University School of Medicine Stanford California USA; ^3^ Center for Biomics Erasmus MC Erasmus University Medical Center Rotterdam Rotterdam The Netherlands

**Keywords:** acetylation, butyrate, histone deacetylase, mast cells, super‐enhancers

## Abstract

Mast cells are key drivers of allergic inflammation. We have previously shown that butyrate, a short‐chain fatty acid derived from dietary fibers, inhibits human mast cell activation and degranulation. Here, we characterized the mechanisms underlying butyrate‐mediated control of mast cell activity. To this end, we assessed the genome‐wide impact of butyrate, a histone deacetylase (HDAC) inhibitor, on the epigenomic control of mast cell gene expression by integrating transcriptome and histone acetylation (H3K27Ac) profiles obtained from butyrate‐treated primary human mast cells. Butyrate affected a selective set of genes and gene regulatory elements in mast cells. Most prominent was the hypoacetylation of promoter regions of highly expressed genes and super‐enhancers controlling key mast cell identity genes. Perturbation of super‐enhancer activity via pharmacological bromodomain inhibition suppressed degranulation of primary human mast cells, evoking repression of key mast cell identity genes that resembled the inhibitory effects of butyrate. Our data indicate that butyrate inhibits human mast cell activity via surprisingly selective targeting of super‐enhancers to regulate the core mast cell transcriptional program.

## Graphical abstract

Butyrate, a short‐chain fatty acid (SCFA) derived from dietary fibers, selectively inhibits human mast cell activation by targeting transcription start sites (TSS) and super‐enhancers regulating key mast cell genes. Local histone hypoacetylation induced by butyrate likely drives transcriptional suppression and reduced degranulation, providing new insights into how SCFA modulates inflammation.

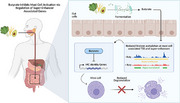

**Abbreviations**
ChIP‐Seq: chromatin immunoprecipitation coupled to high‐throughput sequencing
HAT: histone‐acetyltransferase
HDAC: histone deacetylase
HDACi: histone deacetylase inhibitor
Mb: megabase
MC: mast cell
PBCMCs: peripheral blood–derived cultured mast cells
RNA‐seq: RNA sequencing
SCFA: short‐chain fatty acid
SE: super‐enhancer
SP: substance P
TE: typical‐enhancer
TSS: transcription start site
RPKM: reads per kilobase per million

## 1 Introduction

Mast cells are major effector cells of the immune system. They reside in virtually all vascularized tissues, especially those in direct contact with the external environment. Mast cells mediate IgE‐associated type 2 immune responses, which have been implicated in antiparasite immunity but also allergies, asthma, chronic rhinosinusitis with nasal polyps, and urticaria [1, 2, 3, 4, 5, 6]. Allergens can crosslink allergen‐specific IgE bound to the high‐affinity IgE receptor (FcεRI) on the mast cell surface, resulting in their degranulation^1^. This causes the immediate release of preformed granule mediators such as histamine, heparin, and certain proteases, followed by de novo synthesis and release of various lipid mediators and cytokines. More recently, the Mas‐related G protein‐coupled receptor X2 (MRGPRX2) expressed on connective‐tissue type mast cells was shown to participate in IgE‐independent mast cell activation, resulting in drug‐induced pseudoallergic reactions (e.g., against antibiotics) [7, 8, 9].

Mast cell maturation, phenotype, and function are determined by gene expression programs controlled by endogenous and microenvironmental factors [10]. These include the microbiome, which directly contributes to the development and maturation of the immune system [11]. Short‐chain fatty acids (SCFAs) including butyrate, propionate, and acetate—derived from bacterial fermentation of dietary fibers—are considered key metabolites in the regulation of host physiology and pathophysiology [12, 13, 14]. Butyrate critically affects the differentiation and function of many lymphocyte populations as well as myeloid cells such as macrophages and dendritic cells [15, 16, 17, 18, 19, 20, 21, 22, 23]. In the gut, mast cells are exposed to high concentrations of SCFAs, reaching up to 140 mM in the proximal colon, with butyrate levels at approximately 21 mM [12]. SCFAs are also detectable in the circulation, with concentrations ranging from 1 to 404 µM for total SCFAs and 1 to 64 µM for butyrate [24, 25], and have been previously implicated in protecting against allergic airway inflammation, although the specific role of mast cells in this context has not been investigated [26].

Butyrate is thought to promote gut homeostasis and immunity via control of histone acetylation and subsequently gene transcription [27, 28]. The acetylation state of a given genomic locus is controlled by two classes of antagonistic histone modifying enzymes, histone acetyltransferases (HATs) and histone deacetylases (HDACs), which add and remove target histone acetyl groups, respectively. Histone acetylation is a hallmark of active promoter regions and transcription start sites (TSSs) but also occurs at distal gene regulatory elements such as enhancers [29, 30]. Particularly high levels of histone acetylation are located at super‐enhancers, a class of powerful enhancers that control the expression of key cell type‐specific genes [31]—including in primary human peripheral blood–derived cultured mast cells (PBCMCs) [32]. Butyrate is a known inhibitor of all class I/II HDACs [33, 34] and can significantly affect gene expression since histone acetylation is generally associated with accessible chromatin and active gene transcription [35, 36]. HDAC inhibition is thus expected to trigger histone hyperacetylation and facilitate transcriptional activation. However, whether butyrate affects histone acetylation status and the gene regulatory function of super‐enhancers in mast cells remains largely unknown.

We recently reported that butyrate inhibits human mast cell activation and degranulation, which was associated with reduced expression of genes critical for FcεRI‐mediated signaling—likely via epigenetic mechanisms [37]. How butyrate exerts such specific effects on gene expression in mast cells while targeting a very basal component of transcriptional regulation remains incompletely understood. To address this question, we use extended transcriptome and longitudinal profiling of histone acetylation measurements to identify molecular mechanisms underlying selective gene expression changes in primary human mast cells exposed to butyrate—ultimately resulting in potent mast cell inhibition.

## 2 Results

### 2.1 Butyrate Selectively Regulates Gene Transcription in Primary Human Mast Cells

**FIGURE 1:**
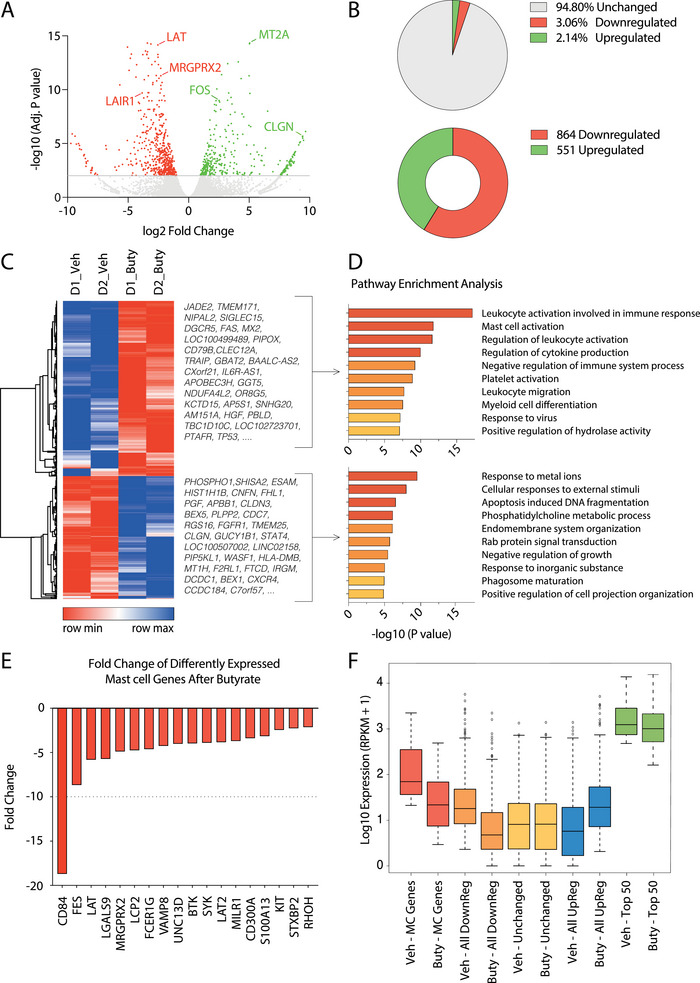
Butyrate selectively downregulates gene expression associated with leukocyte/mast cell activation. Gene expression profiles of primary human mast cells after 24 h of 5 mM butyrate or vehicle treatment were measured using RNA sequencing (RNA‐seq; two biological replicates from independent donors). (A) Scatter plot of downregulated (indicated in red), upregulated (indicated in green), and unchanged genes (indicated in grey) in response to 24 h butyrate treatment. (B) Proportions (*top*) and absolute numbers (*bottom*) of downregulated (red), upregulated (green), and unchanged genes (gray). (C) Heatmap showing scaled expression levels of down‐ and upregulated genes, selected example genes (with the highest fold change) are presented next to the heatmap. (D) Pathway enrichment analysis of downregulated (top) and upregulated (below) genes. (E) Butyrate‐induced downregulation (fold change) of genes associated with the GO:0045576 “Mast Cell Activation” pathway is shown as a bar graph in human mast cells treated with butyrate for 24 h. (F) Expression changes in response to butyrate of the 18 downregulated canonical mast cell genes (red), all 864 downregulated genes (orange), unchanged genes (defined as the 600 genes with the lowest fold change in expression after butyrate treatment, yellow), all 551 upregulated genes (blue), and the top 50 highest expressed genes (green). MC, mast cell; RPKM, reads per kilobase per million. Differentially expressed genes, calculated using DESeq2, were defined as differential genes with an adjusted *p*‐value < 0.05 (Wald test).

To characterize the impact of butyrate exposure on the mast cell transcriptome, we measured gene expression profiles of two independent PBCMCs upon 24 h of 5 mM butyrate or vehicle (IMDM medium) treatment using RNA‐sequencing (RNA‐Seq). The 5 mM concentration was chosen based on previously measured butyrate concentrations in the gut [12] Across all genes detected, 551 were upregulated by butyrate whereas 864 genes were downregulated (FDR < 0.05; Figure 1A,B). The correlation of gene expression values between the two biological replicates was high, both before and after butyrate treatment (*R* ^2^ > 0.97, Figure S1A,B). In line with our previous analyses [37], pathway enrichment analyses indicated that downregulated genes were mainly associated with leukocyte and mast cell activation (Figure 1C,D, upper panels). Indeed, expression of genes coding for proteins involved in FcεRI‐ and MRGPRX2‐mediated mast cell activation was significantly downregulated (including BTK, SYK, LAT, and MRGPRX2; see Figure 1E; Table S1), supporting our earlier findings that butyrate inhibits mast cell activation induced via IgE and substance P (an MRGPRX2 ligand). Upregulated genes were enriched in more diverse biological pathways (Figure 1C,D, lower panels), including genes involved in responses to metal ions (i.e., MT1 family proteins), cellular responses to external stimuli, and Ras/Rab GTPase signaling (Figure 1C,D; Table S1).

The downregulated genes—in particular canonical mast cell genes—showed substantially higher median basal expression values than unaffected or upregulated genes (Figure 1F). This is in line with a previous study in cancer cells demonstrating that HDAC inhibitors are more likely to repress highly expressed genes [26]. Conversely, upregulated genes displayed slightly lower basal expression levels compared with unaffected genes (Figure 1F). Nevertheless, high basal expression levels were not solely predictive of responsiveness to butyrate treatment, as the 50 most highly expressed genes did not respond to butyrate treatment (Figure 1F; green boxplots).

Together, these results show that butyrate, in primary human mast cells, selectively downregulates gene expression associated with leukocyte/mast cell activation and upregulates a more diverse group of genes. Basal expression levels alone only partially predict responsiveness to butyrate treatment, indicating a more selective mechanism of action through which butyrate exerts its effects.

### 2.2 Butyrate Triggers Global Histone Hyperacetylation

**FIGURE 2:**
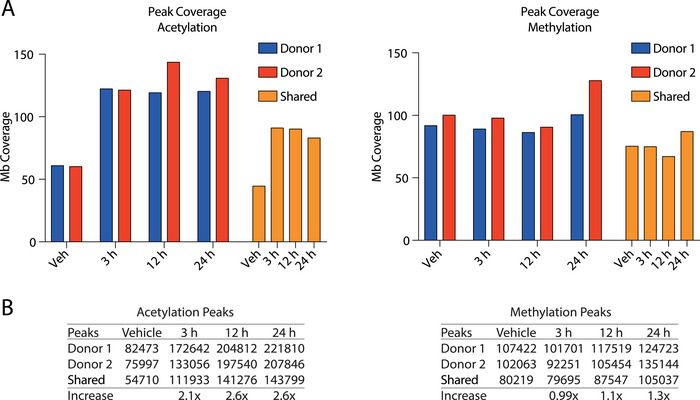
Butyrate triggers global histone hyperacetylation without large‐scale indirect effects on other histone modifications. Chromatin immunoprecipitation (ChIP)‐Seq for histone 3 lysine 27 acetylation (H3K27Ac) and histone 3 lysine 4 di‐methylation (H3K4Me2) was performed after 3, 12, and 24 h of butyrate or 24 h vehicle treatment. (A) Megabase (Mb) coverage of histone acetylation (H3K27Ac, left) and methylation (H3K4Me2, right). Donor 1 is indicated in blue and donor 2 is indicated in red. Overlap between the donors is indicated by the shared peaks (yellow bars). (B) Total number of called acetylation (left) and methylation (right) peaks following butyrate treatment in donor 1 and donor 2, with shared peaks and increases in peak count (based on shared peak counts) indicated.

To map the epigenomic landscape of PBCMCs after butyrate treatment, we extended our previous [37] chromatin immune‐precipitation (ChIP)‐Seq analysis of histone 3 lysine 27 acetylation (H3K27Ac) and added histone 3 lysine 4 dimethylation (H3K4Me2) measurements, obtaining datasets for both histone marks at 0, 3, 12, and 24 h of butyrate treatment. H3K27Ac is a well‐characterized acetylation mark that strongly correlates with transcriptional activity; H3K4Me2 is also associated with active genes yet is not an HDAC target [38]. Genome‐wide H3K27Ac coverage (i.e., positive signals detected above background levels) was markedly increased by butyrate (up to ∼2.6 fold), which was already apparent after 3 h and remained elevated after 24 h of treatment (Figure 2A, left panel). The relative increase in H3K27Ac+ regions across the genome was essentially independent of enrichment calling parameter settings (Figure S2A), and the location of these hyperacetylated regions was largely consistent between different donors (Figure 2A, “Shared” peaks). Butyrate treatment did not affect the overall coverage of H3K4Me2 (Figure 2A, right panel, Figure S2B). In addition to coverage per megabase (Mb) of DNA, butyrate treatment increased the number of individually called H3K27Ac peaks in primary human mast cells ∼2.6‐fold (from 54,710 to 143,799) after 24 h, while H3K4Me2 peaks showed a much more modest increase (∼1.3‐fold, from 80,219 to 105,037 at 24 h) (Figure 2B). Furthermore, butyrate treatment shifted the relative abundance of acetylation at genomic locations from TSS regions (from ∼17% down to ∼10%) to intronic and intergenic regions (Figure S2B).

In conclusion, these data strengthen the notion that butyrate has a profound effect on genome‐wide H3K27Ac histone acetylation dynamics via HDAC inhibition. Notably, these effects occurred without large‐scale indirect effects on other histone modifications such as H3K4Me2.

### 2.3 Distinct Patterns of Histone Acetylation Dynamics Induced by Butyrate

To gain a more quantitative picture of H3K27Ac dynamics upon butyrate treatment, we performed differential enrichment analyses with DESeq2 on reproducible peaks, which showed that most regions acetylated at baseline (>68%) were not significantly (fold change > 2 and adjusted *p*‐value < 0.05) affected by 3–24 h butyrate treatment (Figure S3). After 3 h exposure, butyrate mostly triggered hyperacetylation (6.3% hyperacetylated vs. 2.2% hypoacetylated peaks), whereas 12 h butyrate exposure showed similar proportions of hyper‐ and hypoacetylation at baseline H3K27Ac+ regions (13.3% vs. 12.8%). Finally, 24 h of treatment mostly induced hypoacetylation of existing peaks (13.1% vs. 18.6%, Figure S3).

**FIGURE 3:**
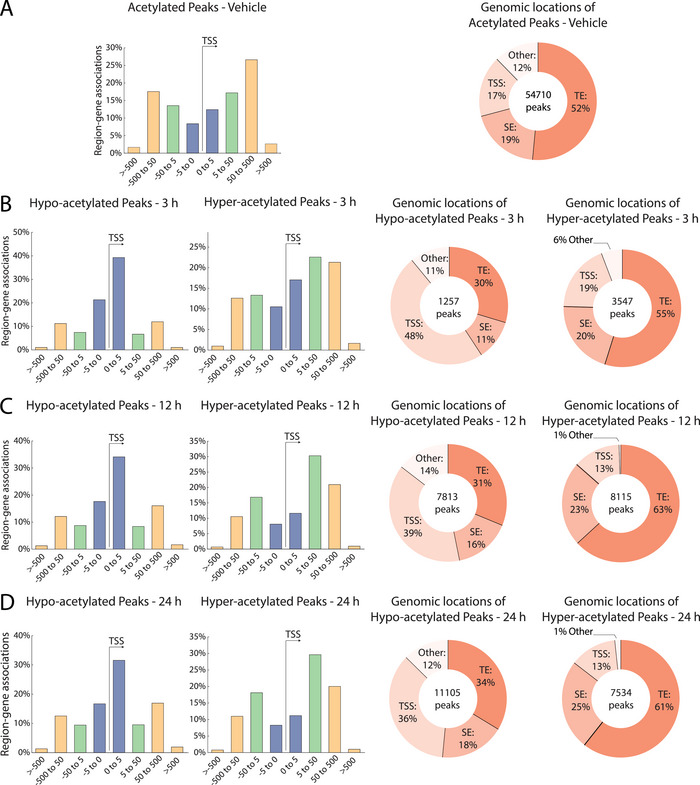
Butyrate induces distinct patterns of histone acetylation dynamics. (A) Distance of acetylated peaks in 24 h vehicle‐treated human mast cells to the associated transcription start site (TSS) regions, both upstream (left side) and downstream (right side). Distances of peak to TSS from 0 to 5 kb are indicated in purple, 5–50 kb in green, and distances greater than 50 kb are indicated in yellow. Donut plots indicate the genomic locations of differentially acetylated regions, which were defined as typical enhancers (TE), super‐enhancers (SE, identified using ROSE), TSS regions (TSS), or others. (B–D) The same analysis as in A but for mast cells treated with butyrate for 3 (B), 12 (C), and (D) 24 h. Differential enrichment was calculated using DESeq2 on reproducible peaks (fold change > 2 and adjusted *p*‐value < 0.05).

To better annotate at which regulatory sites butyrate‐induced histone acetylation dynamics occur, we intersected regions of dynamic acetylation with TSS locations and putative typical enhancer or super‐enhancer regions as defined by the rank ordering of super‐enhancers (ROSE) algorithm [31, 39]. For vehicle‐treated conditions, histone acetylation was primarily located distant from TSS regions, with the bulk of H3K27Ac+ sites located 50–500 kb away from the TSS (Figure 3A, bar graph). Furthermore, ∼52% and ∼19% of acetylated peaks were associated with typical enhancers and super‐enhancers, respectively, whereas 17% of acetylated peaks were associated with TSS regions (Figure 3A, donut graph).

A 3 h exposure to butyrate led to 1226 significantly hypoacetylated peaks, which were predominantly located near TSSs (Figure 3B, within ±5 kb). In fact, ∼48% of all hypoacetylated regions were located at a TSS (Figure 3B, left donut graph). Although the total number of hypoacetylated peaks strongly increased with prolonged butyrate exposure (8.8‐fold increase), their location remained disproportionally enriched at TSS regions (Figure 3B–D). Oppositely, butyrate treatment for 3 h led to 3547 significantly hyperacetylated peaks, which were predominantly located distal of TSS regions (Figure 3B, >5 kb upstream or downstream). Overall, ∼55% of all hyperacetylated regions intersected with a typical enhancer, and ∼21% intersected with a super‐enhancer (Figure 3B, right donut graph). Although the number of hyperacetylated peaks increased with prolonged butyrate exposure (2.1‐fold increase), their location remained disproportionally enriched at enhancer locations (Figure 3B–D). Specifically, 12 and 24 h after butyrate treatment, ∼86% of all hyperacetylated peaks were located either at a typical enhancer or super‐enhancer (Figure 3C,D).

These data reveal that butyrate—despite its potent capacity for HDAC inhibition—affects histone acetylation at a relatively small subset (i.e., ∼30%) of pre‐existing H3K27Ac+ chromatin regions. Strikingly, butyrate‐induced HDAC inhibition preferentially reduces H3K27Ac levels at TSSs, while H3K27Ac enrichment primarily occurs at locations distal from TSS regions, co‐localizing with typical enhancers and super‐enhancers.

### 2.4 Differential Impact of Butyrate on Acetylation Dynamics at Transcriptionally Activated or Repressed Loci

**FIGURE 4:**
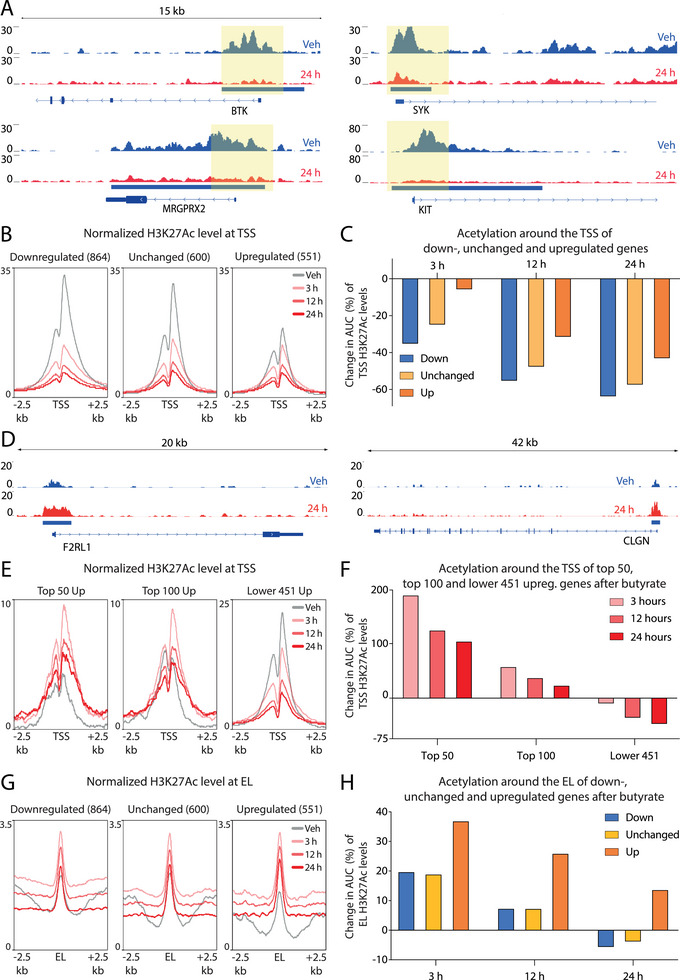
Butyrate exposure induces complex histone acetylation dynamics that only partially correlate with transcriptional outcomes. RNA‐Seq and ChIP‐Seq datasets, obtained from butyrate‐treated primary human mast cells derived from two different donors, were integrated to investigate the relationship between butyrate‐mediated controls of histone acetylation and gene expression. (A) Representative examples of hypoacetylated (indicated by a blue rectangle) canonical mast cell genes (i.e., *BTK*, *SYK*, *MRGPRX2*, and *KIT*). Yellow shading highlights significant differences between histone acetylation in vehicle‐treated human mast cells (indicated in blue) and butyrate‐treated human mast cells (indicated in red, 24 h). (B) Histograms of H3K27Ac at the TSS of downregulated genes (left box), unchanged genes (middle box), and upregulated genes (right box). The vehicle condition is indicated in light grey and TSS acetylation after butyrate treatment is indicated in shades of red. (C) Quantification of the reduction of TSS acetylation area under the curve at 3, 12, and 24 h after butyrate treatment (with downregulated genes indicated in blue, unchanged genes in yellow, and upregulated genes in orange). (D) Examples of hyperacetylated genes (i.e., *F2RL1* and *CLGN*). (E) Histograms of H3K27Ac at the TSS of the top 50 upregulated genes (left box), top 100 upregulated genes (middle box), and lower 451 upregulated genes (right box). (F) Area under the curve quantification of data shown in E. (G) Histograms of H3K27Ac at the enhancer landscape (EL) of downregulated genes (left box), unchanged genes (middle box), and upregulated genes (right box). (H) Area under the curve quantification of data shown in G. Differential enrichment was calculated using DESeq2 (fold change > 2 and adjusted *p*‐value < 0.05).

We next assessed how the observed changes in histone acetylation upon butyrate treatment translate into altered gene expression. We observed hypoacetylated TSS peaks near several downregulated genes, including the mast cell activation‐associated genes BTK (3.97‐fold decrease expression), SYK (3.89‐fold), MRGPRX2 (4.86‐fold), and KIT (2.45‐fold) (Figure 4A). Overall acetylation at the TSS of the 864 (Figure 1B) downregulated genes was strongly and immediately reduced upon butyrate exposure (Figure 4B, left histogram). Although TSS acetylation was also reduced at transcriptionally unchanged genes (defined as the 600 genes with the lowest fold change in expression after butyrate treatment) and the 551 upregulated genes (Figure 4B, middle and right histogram), the quantitative reduction in area under the curve was strongest and fastest for the 864 downregulated genes (Figure 4C).

We also tested whether increased histone acetylation upon butyrate treatment was linked to elevated gene expression levels. Several strongly upregulated genes (Log2 fold change > 4) displayed hyperacetylation at their TSS regions (representative examples are F2RL1 and CLGN, which show 1500‐ and 675‐fold increased expression, respectively; Figure 4D). Indeed, acetylation at the TSS of the 50 most upregulated genes was increased at all measured timepoints (Figure 4E, left histogram). Acetylation at the TSS of a broader set of the 100 most upregulated genes was also increased at 3 h after butyrate exposure, although H3K27Ac levels returned to near basal levels after 12 and 24 h (Figure 4E, middle histogram). However, the 451 remaining upregulated genes, which showed weaker induction and higher baseline TSS acetylation, showed reduced TSS H3K27Ac signals in response to prolonged (>3 h) butyrate treatment (Figure 4E, right histogram). The area under the curve quantification validated that acetylation at the TSS of strongly upregulated genes rapidly increased, whereas acetylation at the TSS of moderately or weakly upregulated genes actually decreased (Figure 4F). Notably, the most strongly upregulated genes (i.e., top 50/100) displayed lower baseline acetylation (Figure 4E) and RNA expression (Figure S4), whereas less strongly upregulated genes had established TSS acetylation and higher expression prior to butyrate treatment (Figure S4). These results suggest that transient hyperacetylation at the TSS of genes may be sufficient to induce gene activation at poorly expressed or silent genes, whereas the butyrate‐mediated induction of already robustly expressed genes is regulated by TSS acetylation‐independent mechanisms.

As the majority of hyperacetylated peaks were located at typical enhancers (Figure 3B–D), we next quantified changes in acetylation enrichment in the enhancer landscape (EL, defined as all non‐TSS H3K27Ac peaks). Histone acetylation at the EL linked to the 864 downregulated and unchanged genes displayed an initial modest increase but decreased again after 24 h (Figure 4G, left and middle histogram). Acetylation at the EL around the 551 upregulated genes was increased at all measured timepoints (Figure 4G, right histogram), which was supported by quantification of the area under the curve (Figure 4H).

Taken together, these findings demonstrate that butyrate exposure induces complex histone acetylation dynamics. Generally, H3K27Ac was rapidly depleted around the TSS of genes, which was most pronounced for downregulated genes. Only very strongly induced genes expressed at very low baseline levels did show a sustained gain in promoter H3K27Ac levels. The overall effects on the enhancer landscape appear to be more modest, although hyperacetylation of enhancers may, in part, be linked to the upregulated expression of associated genes.

### 2.5 Butyrate Preferentially Suppresses Super‐Enhancers and Their Associated Genes

**FIGURE 5:**
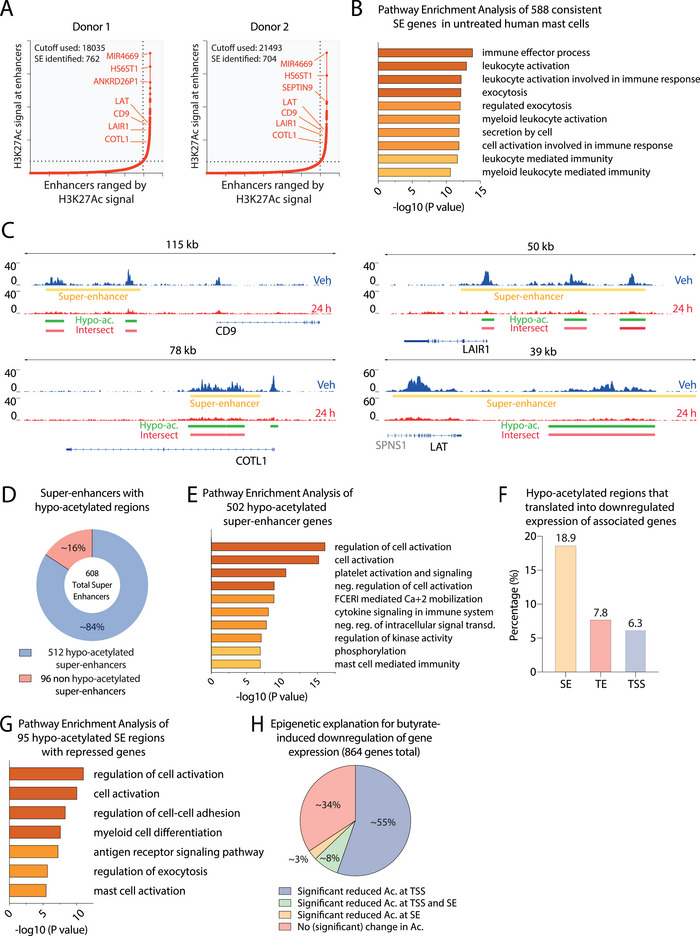
Butyrate perturbs super‐enhancers and preferentially suppresses SE‐associated transcripts. (A) Super‐enhancers (SE) were ranged by H3K27Ac signal in vehicle‐treated human mast cells of donor 1 (left hockey stick graph) and donor 2 (right hockey stick graph). (B) Pathway enrichment analysis of 588 consistent (present in both donor 1 and 2) SE genes in vehicle‐treated human mast cells. (C) Representative examples of SE (indicated by yellow rectangles) in vehicle‐treated human mast cells (indicated in blue), that become hypoacetylated (indicated by green bars) in response to butyrate treatment (24 h, indicated in red). Overlap between identified SE and hypoacetylated regions is indicated by red bars (intersect). (D) The proportion of SE that contains hypoacetylated regions (in blue) and SE regions that do not intersect with a hypoacetylated region (in light red). (E) Pathway enrichment analysis of 502 hypoacetylated SE genes. (F) The percentage of hypoacetylated SE, typical enhancer (TE), or TSS regions linked to a downregulated gene. (G) Pathway enrichment analysis of 95 downregulated genes associated with hypoacetylated SE regions. (H) Epigenetic explanation for butyrate‐induced downregulation of gene expression. The proportion of downregulated genes with significant hypoacetylated TSS (purple), TSS and SE (green), SE (yellow), and nonsignificant reduced acetylation (pink). Differential enrichment was calculated using DESeq2 (fold change > 2 and adjusted *p*‐value < 0.05).

Because super‐enhancers can be extensively acetylated and have been reported as preferential targets of HDAC inhibitors [40, 41], we next investigated H3K27Ac dynamics at super‐enhancers and transcriptional changes of their target genes. From two biological replicates, 608 reproducible super‐enhancers were identified that were linked to 588 unique associated genes in vehicle‐treated primary human mast cells (Figure 5A). These genes were strongly enriched for immune effector cell processes, such as cell activation, exocytosis, and secretion (Figure 5B). These included super‐enhancers associated with *CD9*, *LAIR1*, *COTL1*, and *LAT*, which are known regulators of mast cell mediator secretion (Figure 5C), as well as other mast cell‐associated genes such as *KIT*, *MS4A2*, and *LYN* (Table 2S).

Most super‐enhancers (∼84%) overlapped with non‐TSS regions that were hypoacetylated (fold change > 2 and adjusted *p*‐value < 0.05) after 24 h of butyrate treatment (Figure 5C,D). Pathway enrichment analyses of the 502 genes associated with hypoacetylated super‐enhancer revealed that they are particularly associated with the regulation of cell activation, FCERI‐mediated Ca^+2^ mobilization, and mast cell‐mediated immunity (Figure 5E). Of note, ∼19% of hypoacetylated super‐enhancers were linked to transcriptionally downregulated genes (Figure 5F), as compared with only 6–8% for hypoacetylated typical enhancers and TSS regions. For genes expressed prior to butyrate treatment (RPKM>1), hypoacetylation of super‐enhancers represented the strongest predictor of transcriptional downregulation (24%), followed by hypoacetylation of typical enhancers (13.0%) and TSS regions (7.7%) (Figure S5A).

Genes that displayed downregulated expression and super‐enhancer hypoacetylation (*n* = 95, 18.9% of 502 genes) were strongly associated with mast cell activation and exocytosis (Figure 5G). By contrast, downregulated genes linked to hypoacetylated typical enhancer regions are associated with more general immune functions (Figure S5B). Together, these findings provide a plausible epigenetic explanation for the transcriptional deregulation of many downregulated genes, that is, via loss of histone acetylation of their TSSs, super‐enhancers, or both (Figure 5H). Of note, out of the 864 downregulated genes, 112 genes were associated with super‐enhancers, a majority (85%) of which displayed hypoacetylation after 24 h of butyrate.

A considerable share of hyperacetylated peaks was located in super‐enhancer regions (∼21–25%) (Figure 3B–D), and ∼20% of super‐enhancers contained a hyperacetylated region after 24 h of butyrate treatment (Figure S5C). Yet, only ∼0.4% of hyperacetylated super‐enhancers were linked to transcriptionally upregulated genes, similar to hyperacetylated typical enhancers and TSS regions (Figure S5D). Instead, weak hyperacetylation (i.e., not passing our thresholds for differential enrichment) at typical enhancers may better explain why expression is induced at such genes (Figure S5E,F).

In summary, these data indicate that super‐enhancers are an important target of butyrate‐induced HDAC inhibition, most often resulting in a loss of H3K27Ac and correlating with reduced expression of many associated key mast cell genes.

### 2.6 Human Mast Cell Activation Is Regulated by Super‐Enhancer Activity and the Associated Cell‐Type Specific Transcriptional Networks

**FIGURE 6:**
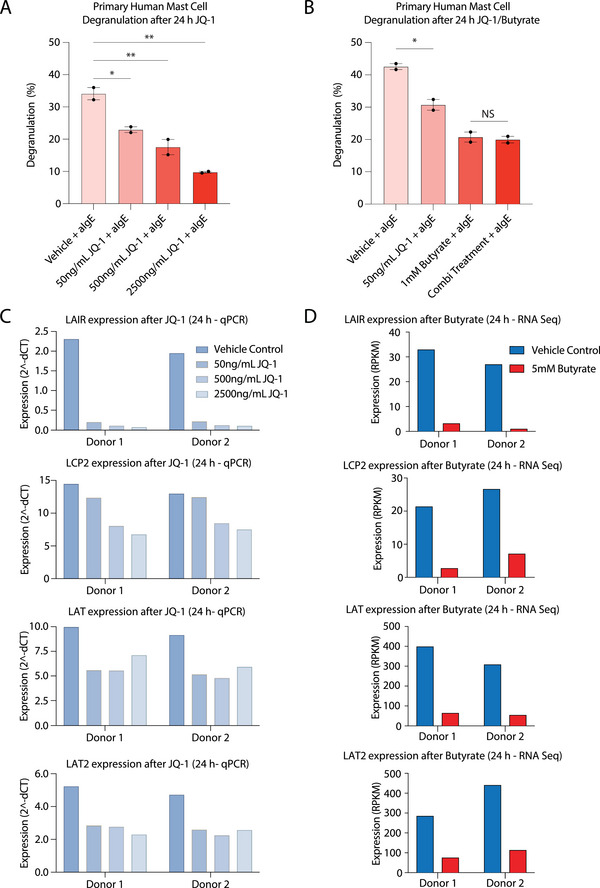
BET‐bromodomain inhibitor JQ‐1 inhibits human mast cell activation to a similar extent as butyrate. (A) Primary human mast cells were treated with increasing concentrations of JQ‐1 for 24 h, primed with IgE, and cross‐linked with anti‐IgE. Percentage of mast cell degranulation (as measured by beta‐hexosaminidase release) after IgE‐mediated activation using anti‐IgE stimulation in primary human mast cells treated with increasing concentrations of JQ‐1. (B) Percentage of degranulation after IgE‐mediated human mast cell activation in vehicle‐treated, JQ‐1 treated (50 ng/mL, 24 h), butyrate‐treated (1 mM, 24 h) or combination‐treated (JQ‐1+butyrate, 24 h) cells. (C) Gene expression (2^delta CT) of *LAIR*, *LCP2, LAT*, and *LAT2* in human mast cells derived from two different donors treated with increasing concentration of JQ‐1 for 24 h. (D) Gene expression (RPKM) of *LAIR*, *LCP2, LAT*, and *LAT2* in human mast cells derived from two different donors treated with 5 mM Butyrate or vehicle for 24 h. Data represent mean ± SD (panels A and B), and statistical significance was tested using a one‐way ANOVA test: *Significantly decreased compared with vehicle control (*p* < .05). ***p < .001. NS, not significant. Data were obtained from human mast cells derived from two different donors, with each condition representing an average of 2–3 technical replicates. Data represent mean ± SEM, statistical significance was tested using a one‐way ANOVA test.

Next, we set out to assess whether specific perturbation of super‐enhancer activity can indeed affect human mast cell activation. To this end, we treated primary human mast cells with JQ‐1, a bromodomain containing 4 (BRD4) inhibitor, and induced degranulation by IgE/antigen stimulation. BRD4 was found highly enriched at super‐enhancer regions in all cell types tested to date, regulating the expression of associated genes [31, 39, 42, 43, 44]. JQ‐1 potently inhibited primary human mast cell degranulation, in a concentration‐dependent manner (Figure 6A). JQ‐1 did not induce cell death at the tested concentrations, with cell viability remaining ∼94% in both vehicle and JQ‐1 treated cells (Figure S6A). To assess whether the effects of JQ‐1 were (non‐)additive to the effects of butyrate on human mast cell activation, suboptimal concentrations of JQ‐1 (50 ng/mL) and butyrate (1 mM), as well as a combination of the two inhibitors, were incubated with the cells for 24 h followed by IgE crosslinking. Inhibition of mast cell degranulation by butyrate was not further increased by (low‐dose) JQ‐1 addition, suggesting that both inhibitors target the same modulators of human mast cell activation (Figure 6B). Indeed, JQ‐1 diminished the expression of super‐enhancer‐associated genes *LAIR*, *LCP2*, *LAT*, and *LAT2* (Figure 6C), which were also repressed by butyrate (Figure 6D). In line with the nonadditive effect of JQ‐1 on inhibition of degranulation by butyrate, JQ‐1 also did not exert significant additive effects on transcriptional repression of super‐enhancer‐associated genes by butyrate (Figure S6B). Since LAIR, LCP2, LAT, and LAT2 are essential modulators of human mast cell activation [45, 46, 47, 48], these observations may explain why mast cells display an inhibited degranulation profile after both butyrate and JQ‐1 exposure.

Given that 1 mM of butyrate already induced repression of various super‐enhancer genes (Figure S6B), we set out to assess whether prolonged exposure to this concentration would remain an effective means to suppress mast cell activity (Figure S6C). We observed that a single 1 mM dose of butyrate still substantially decreased mast cell degranulation and IL‐8 production after 7 days. However, these effects were ultimately transient, subsiding after 14 days (Figure S6C).

A simple explanation for preferential deacetylation at TSS and super‐enhancer regions after butyrate treatment may be a nonselective redistribution of histone acetylation that affects such regions more profoundly due to their extensively acetylated nature. However, a substantial number of established highly enriched H3K27Ac peaks either increased in acetylation levels or—even more frequently—were unaffected by butyrate treatment (representative examples in Figure S6D). For example, only ∼31% of the 500 most extensively acetylated sites in the mast cell genome were hypoacetylated by butyrate after 3 h (Figure S6E). Nevertheless, most hypoacetylated peaks (∼74%; 890 out of 1203) after 3 h treatment originated from the 5000 strongest H3K27Ac peaks (Figure S6F). Thus, butyrate‐induced hypoacetylation selectively targets a subset of extensively acetylated peaks.

Taken together, these data indicate that human mast cell activation is strongly regulated by super‐enhancer activity and the associated cell‐type specific transcriptional networks. Furthermore, butyrate likely exerts (part of) its inhibitory effects on human mast cell activation via the specific perturbation of super‐enhancer activity.

## 3 Discussion

Although we and others have demonstrated that butyrate and other HDAC inhibitors can regulate the activity of mast cells and other immune cells, it remains incompletely understood how such broadly acting molecules can exert rather precise and cell‐type specific transcriptional changes. By integrating RNA‐Seq and ChIP‐Seq datasets obtained from butyrate‐treated primary human mast cells, we uncovered that butyrate controls mast cell function through complex yet highly specific changes in histone acetylation—including a loss of acetylation at super‐enhancers that control key mast cell degranulation genes. Pharmacological inhibition of super‐enhancers indeed suppressed mast cell degranulation, similar to what was observed for butyrate.

We found that gene repression following butyrate treatment is primarily associated with H3K27Ac depletion around the TSS of highly expressed genes. Although seemingly counterintuitive, many studies have reported gene repression triggered by HDAC inhibitors [49]. Highly expressed genes were previously shown to be prime targets of HDAC inhibitors in (cancer) cell lines [50, 51], including KIT in transformed human mast cells [52], in line with our own findings. This may be explained by preferential binding of HDACs to highly expressed genes, where they are considered to be critical for maintaining the precise acetylation‐deacetylation balance required for productive gene transcription [51, 53]. As histone acetylation attracts many proteins involved in transcriptional control, global redistribution of histone acetylation, as caused by butyrate, is likely to redirect these chromatin readers away from their target regulatory regions [51]. While our findings agree with these notions, we also show that many highly expressed and strongly acetylated genes are completely impervious to HDAC inhibition by butyrate, indicating that additional mechanisms determine the remarkable selectivity by which butyrate affects the epigenome and transcriptome of mast cells.

In agreement with findings by Rada‐Iglesias et al. [54], our analysis of H3K27Ac dynamics in mast cells revealed that butyrate‐induced hyperacetylation of existing H3K27Ac+ sites is mostly an early and transient event since hypoacetylation became the dominant effect at 24 h. This suggests that histone deacetylation might be a secondary effect of HDAC inhibition, as HATs gain a competitive advantage for acetyl groups and are able to deposit these at new locations. In primary human mast cells, loss of H3K27Ac at gene regulatory regions upon butyrate treatment was not caused by notable reductions in HAT expression (data not shown). Important to note here is that a previous study of the HDAC inhibitor largazole revealed that exposure to low concentrations solely induced gene activation, whereas higher concentrations shifted the balance toward gene repression [55]. Importantly, it has been shown that both local and systemic concentrations of butyrate are influenced by various external conditions. These include: (1) dietary intake, for example, fecal butyrate concentrations increased from ∼11 to ∼16 µmol/g after consumption of arabinoxylan‐oligosaccharides enriched bread in healthy volunteers [56, 57], and pig hepatic vein butyrate levels increased from ∼6 to ∼17 µM after receiving an arabinoxylan‐enriched diet [57]); (2) disease conditions, for example, fecal butyrate concentrations from inflammatory bowel disease patients were lower (93 µmol/g) than in healthy subjects (176.0 µmol/g) [58]; and (3) antibiotic treatment, for example, mouse cecal butyrate concentrations dropped from 10 to 0.01 mM after streptomycin treatment [59]). Such fluctuations in butyrate levels need to be carefully considered, as they may significantly affect mast cell function and immune responses in vivo.

To the best of our knowledge, our study provides the first in‐depth characterization of super‐enhancers and their associated genes in primary human mast cells. Similar to our approach, Cildir et al. [32] ranked enhancers in human mast cells (PBCMCs) based on H3K27Ac levels, generating a list of super‐enhancers. However, the authors did not further investigate the functional roles specifically associated with super‐enhancers and linked genes [32], as opposed to transcriptional regulation by conventional enhancers. We found that super‐enhancer‐associated genes in human mast cells were strongly linked to immune effector cell processes, such as cell activation, exocytosis, and secretion. Likely due to their high HDAC and HAT occupancy, ∼84% of super‐enhancers contained hypoacetylated regions after butyrate treatment, which correlated with reduced transcriptional output of many nearby genes. Among these affected super‐enhancer‐associated genes are many core regulators of mast cell identity and function, including the KIT receptor, FceRI signaling components, and degranulation‐associated factors. Interestingly, perturbation of super‐enhancer activity by JQ‐1 inhibited degranulation of primary human mast cells, most likely via downregulation of various key mast cell identity genes that are also targeted for repression by butyrate. Indeed, specific repression of core cell identity gene expression by HDAC inhibitors has been previously reported [55, 60]. Importantly, previous studies have demonstrated that BRD4 is strongly enriched at super‐enhancers across various cell types, supporting our use of a BRD4 inhibitor to target super‐enhancer activity in human mast cells [31, 39, 42–44]. Thus, it appears plausible that at least part of the inhibitory effect of butyrate is a direct consequence of super‐enhancer destabilization. How mast cell activation subsequently reorganizes the chromatin landscape, a phenomenon recently described by Cildir et al. [32], and which specific (combination of) repressed genes form the foundation of butyrate's inhibitory effects, are important topics for future studies. It is important to note that while hypoacetylation of super‐enhancers correlated with the repression of key mast cell genes, a larger fraction of genes downregulated by butyrate showed TSS depletion of H3K27Ac levels. Hence, it is plausible that diminished mast cell activation after butyrate exposure is the result of combined targeting of TSS and (super‐)enhancer elements.

In summary, our data indicate that butyrate inhibits mast cell activation via a surprisingly selective suppression of the core mast cell transcriptional program, in part by targeting super‐enhancers. Acquiring a deeper understanding of the mechanisms of action of butyrate, and other HDAC inhibitors, may in the future offer improved ways to combat mast cell‐mediated diseases such as allergies and asthma.

### 3.1 Data Limitations and Perspectives

Our experiments were conducted with relatively high concentrations of butyrate (i.e., 1–5 mM), modeling the exposure levels that mast cells would encounter in the human gastrointestinal tract. Future research should focus on the effects of (prolonged) exposure to physiological SCFA levels beyond those found in the gut, as this may reveal critical insights into the broader role of butyrate in modulating immune responses. A limitation of our study is that while we did not observe additive effects of (suboptimal) JQ‐1 concentrations on the suppressive capacities of butyrate, we cannot completely rule out potential additive effects that may emerge at other time‐points or concentrations of co‐exposure. Additionally, our study did not consider any nonhistone deacetylation effects that butyrate may exert, and which may contribute to the regulation of mast cell activation. Understanding these mechanisms will be essential for elucidating the complex interactions between butyrate and immune cell function.

Notably, we found that a single dose of 1 mM butyrate induced substantial repression of super‐enhancer gene expression, mast cell degranulation, and inflammatory mediator production after a prolonged exposure period (i.e., 7 days). In this context, it will be of significant interest to conduct more extensive studies into potential mechanisms of resistance or adaptation after an initial butyrate exposure.

## 4 Methods

### 4.1 Peripheral Blood–Derived Cultured Mast Cells

Primary human PBCMCs were generated as previously described by Folkerts et al. [37] Briefly, peripheral blood mononuclear cells were obtained from buffy coats of healthy blood donors and CD34^+^ precursor cells were isolated using the EasySep Human CD34 Positive Selection Kit (STEMCELL Technologies, 17856). CD34^+^ cells were maintained for 4 weeks under serum‐free conditions using StemSpan medium (STEMCELL Technologies, 09650) supplemented with recombinant human IL‐6 (50 ng/mL; Peprotech, 200–06), human IL‐3 (10 ng/mL; Peprotech, 200–03), and human Stem Cell Factor (100 ng/mL Peprotech, 300–07). Thereafter, the cells were maintained in IMDM Glutamax I (Thermo Fisher Scientific, 31980030–500 mL), containing sodium pyruvate (Gibco, 11360‐039), supplemented with 0.1% 2‐mercaptoethanol (Thermo Fisher Scientific, 21985023), 0.5% BSA (Sigma‐Aldrich, A9647), 1% insulin‐175 transferrin selenium (Thermo Fisher Scientific, 41400045), Ciprofloxacin (10 µg/mL; Sigma‐Aldrich, 17850), IL‐6 (50 ng/mL; Peprotech), and human Stem Cell Factor (100 ng/mL; Peprotech). After 8–12 weeks, PBCMCs were tested for maturity by Giemsa or toluidine blue staining and beta‐hexosaminidase release assays. Cell viability, both with and without JQ‐1 treatment, was evaluated after 24 h. Briefly, 10 µL of cell suspension was mixed with 10 µL of 0.4% Trypan Blue Stain (Gibco, 15250‐061). The mixture was then placed in a Countess cell counting chamber slide (Thermo Fisher, C10283) and analyzed using the Countess II Automated Cell Counter (Thermo Fisher).

### 4.2 RNA‐Seq Gene Expression Analysis and Pathway Enrichment Analyses

For each experiment and timepoint, a fresh sodium butyrate (Sigma‐Aldrich, 303410) solution was prepared. In brief, 6.88 mg butyrate was dissolved in 2.5 mL IMDM (Gibco, 31980030), to reach a 25 mM concentration, which was further diluted in IMDM and/or cell culture to achieve the desired concentration. Accordingly, 24 h IMDM was used as vehicle treatment. To prepare RNA samples for RNA‐seq, total RNA was isolated from human PBCMCs treated with 5 mM butyrate (or vehicle) for 24 h, using the RNeasy Micro Kit (Qiagen, 74004). High‐throughput sequencing was performed on the Illumina HiSeq 4000 sequencer. Reads were generated of 50 base pairs in length and alignment was performed using HISAT (Hierarchical Indexing for Spliced Alignment of Transcripts). Tag directories were generated for each sample with the removal of duplicate reads (‐tbp 1 option). Quantification and normalization of the RNA‐Seq data was performed using the open‐source software HOMER [61]. Differential expression was calculated using DESeq2 within the environment of HOMER. Significant differentially expressed genes were defined as differential genes with an adjusted *p*‐value < 0.05 (Wald test). To filter out significant differences among lowly expressed genes, an average RPKM value higher than 1 in at least one condition was required. The remaining genes all had a Log2 fold change higher than (−)0.8, we tolerated this cut‐off for further downstream analyses. Pathway enrichment analysis was done using Metascape [62].

### 4.3 ChIP‐Seq and Data Analysis

ChIP was performed as previously described [63], with minor modifications. Per ChIP, 100K crosslinked mature primary human mast cells were used. Sonicated chromatin was immunoprecipitated using 1 µg of anti‐H3K27Ac antibody (Abcam, Ab4729), 1 µg of anti‐H3K4Me2 antibody (Abcam, Ab32356), and 25 µL BSA‐blocked Protein A agarose beads (Millipore #16‐125). Illumina sequencing libraries were prepared using the ThruPLEX DNA‐Seq Kit (Rubicon Genomics, R400676) and sequenced on an Illumina HiSeq2500 sequencer (single read 50 bp length, 17–21 million reads per sample). H3K27Ac ChIP‐Seq data for the 0 and 3 h timepoints for previously generated [37] and re‐analyzed in this study. Reads were aligned to the human GRCh38 genome build using HISAT2 [64] with standard parameters and parsed to HOMER [61] for downstream analyses. Tag directories were generated for each sample with the removal of duplicate reads (‐tbp 1 option). BedGraph files displaying normalized counts (reads per million) were generated for direct visualization in the UCSC Genome Browser using the makeUCSCfile HOMER script. H3K27Ac enriched regions were identified using HOMER findPeaks with ‐region ‐size 150 ‐minDist 370 (parameter set 1) or ‐region ‐size 1000 ‐minDist 2500 (parameter set 2) options. Histograms of ChIP signals were generated with the annotatePeaks script (using the ‐hist option). ChIP‐Seq datasets were deposited in the Gene Expression Omnibus (GSE279725). Differential peaks were calculated using DESeq2 with default settings (fold change > 2 and adjusted *p*‐value < 0.05). Intersect peak files of two different peak files were analyzed via the Genomic Regions Enrichment of Annotations Tool (GREAT) to obtain the distance to associated TSS data. Genomic locations of (differentially) acetylated regions were annotated using the *annotatePeaks.pl* function in HOMER, which provides genomic feature and gene association information.

### 4.4 Analysis of Super‐Enhancers and Typical Enhancers

The ROSE algorithm was utilized to identify super‐enhancers and typical enhancers [31, 39]. Initially, H3K27ac ChIP‐seq peaks within 12.5 kb of each other were stitched together. Regions 2.5 kb upstream and downstream of known transcription start sites were excluded to avoid promoter regions contributing to super‐enhancer identification. The aggregated H3K27ac read counts within these stitched regions were plotted in a ranked order based on their signal intensity. Super‐enhancers were defined as those regions exhibiting exceptionally high levels of H3K27ac signal, situated above the inflection point (defined by the ROSE algorithm) in the ranked list. Conversely, enhancers below this inflection point were classified as typical enhancers. Annotation of these enhancers, pathway enrichment analysis, and differential peak analysis were performed as described above. Super‐enhancers spanning TSS regions were retained, and those overlapping differentially enriched peaks were subsequently removed in downstream experiments.

### 4.5 Mast Cell Activation Assay, Quantitative Real‐Time PCR & ELISA

Mast cells were cultured (if applicable) with JQ‐1 (MedChemExpress, HY‐13030) or vehicle (DMSO, Thermo Fisher, D12345) and/or sodium butyrate (Sigma‐Aldrich, 303410) or vehicle (IMDM, Gibco, 31980030) 24 h prior to activation. PBCMCs from two different donors were sensitized with human IgE (Sigma‐Aldrich, 401152, 2 µg/mL, for 1 h) and washed, followed by stimulation with 2 µg/mL of rabbit anti‐IgE (Fortis Life Sciences, A80‐109A). Degranulation was measured by the release of beta‐hexosaminidase [65]. Substrate 4‐MUG (Sigma‐Aldrich, M2133) was added to this enzyme containing supernatant and the product was measured after 1 h by means of fluorescence (Glomax Discover, Promega). After RNA isolation as stated above, cDNA was made using RevertAid H Minus First Strand cDNA synthesis kit (Thermo Fisher Scientific, #K1632) according to the manufacturer. qPCR is performed using PowerUp SYBR Green Master Mix (Thermo Fisher Scientific, #A25741) and primers were found by OriGene (Table 1). Run on QuantStudio 3 Real‐Time PCR System (Thermo Fisher Scientific, A28567). For the prolonged butyrate exposure studies, mast cells were treated with a single administration of 1 mM butyrate or vehicle for 7–14 days and activated as described above. To determine cytokine production and secretion, supernatant was collected 24 h after mast cell stimulation. Concentrations of human mast cell IL‐8 were determined using an IL‐8 ELISA kit (BioTechne, D8000C) per the manufacturer's protocol.

**TABLE 1:** Quantitative real‐time PCR primers.

Gene name	Forward	Reverse	Gene ID OriGene
Lair1	TGGTCTGAGCAGAGTGACTACC	GCTCATTGTGACTGTTGTCCGAC	3903
LCP2	GGAAGAAGCCACCTGTGCCAAA	GCTCATAGGAAGTAGTGCTGGC	3937
Lat	ATCCTGGAGCGGCTAAGACTGA	GTTCAGCTCCTGCAGATTCTCG	27,040
Lat2	GCAAGCAGAAAACCACAGAGACA	AGAGGGACAGAGACCAGAAGTG	7462
HPRT	ATTGTAATGACCAGTCAACAGGG	GCATTGTTTTGCCAGTGTCAA	In‐house

### 4.6 Statistical Analysis

Statistical tests were performed with GraphPad Prism 9 (GraphPad Software, Inc). One‐way ANOVA tests were performed as described in the respective figure legends. A *p*‐value of less than 0.05 was considered statistically significant.

## Author Contributions

Jelle Folkerts performed the experiments with the technical assistance of Marjolein J. W. de Bruijn and Wilfred F. J. van IJcken. Jelle Folkerts performed data analysis. Rudi W. Hendriks and Ralph Stadhouders supervised the project and participated in experimental design and technical discussions. Jelle Folkerts wrote the first draft of the paper. Jelle Folkerts, Rudi W. Hendriks, and Ralph Stadhouders drafted the final manuscript which all co‐authors was approved.

## Conflicts of Interest

The authors declare no conflicts of interest.

## Peer Review

The peer review history for this article is available at https://publons.com/publon/10.1002/eji.202451680.

## Supporting information




**Supporting file 1**: eji6001‐sup‐0001‐SuppMat.pdf.

**Supporting file 2**: eji6001‐sup‐0002‐TableS1.xlsx.

**Supporting file 3**: eji6001‐sup‐0003‐TableS2.xlsx.

## Data Availability

The data that support the findings of this study are openly available in “Comprehensive Gene Expression and Epigenetic Profiling of Human Mast Cells: Effects of Butyrate Treatment” at https://www.ncbi.nlm.nih.gov/geo/query/acc.cgi?acc=GSE279725, reference number “Series GSE279725”.
