# Supplementary material for: Butyrate Selectively Targets Super‐Enhancers and Transcriptional Networks Associated with Human Mast Cell Function
Source: Eur J Immunol. 2025 Jun 11;55(6):e51680. doi: 10.1002/eji.202451680 (PMC12154173; doi:10.1002/eji.202451680)
Supplement: Supplementary file 1 — Supporting file 1: eji6001‐sup‐0001‐SuppMat.pdf. [file EJI-55-e51680-s003.pdf]

## Folkerts et al. – Supporting Information

**Supplementary Table 1** – RNA-Seq data Vehicle (IMDM medium) vs. 24h butyrate treatment

**Supplementary Table 2** – Human mast cell super-enhancers

**Supplementary Figures 1-6** (see below)

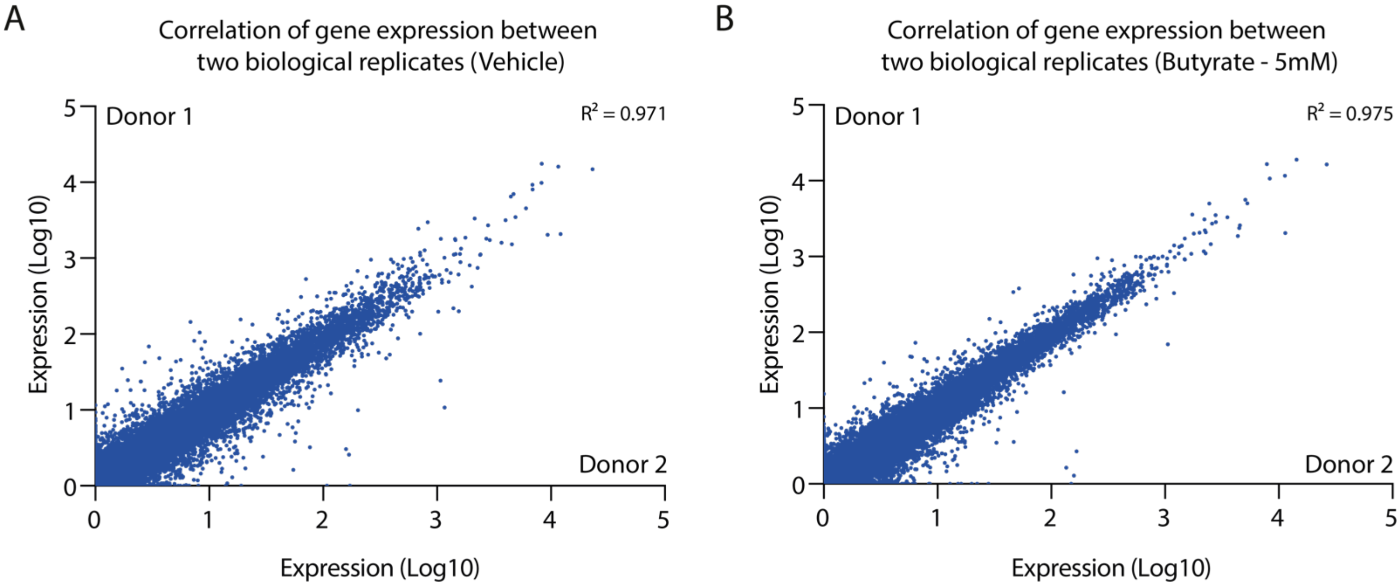


**Supplementary Figure 1. Correlation of gene expression values between both primary human mast cell cultures.** (**A**, **B**), Comparison of gene expression (Log10) profiles between donor 1 and donor 2, in vehicle treated (left scatter plot) and butyrate treated human mast cells (right scatter plot).

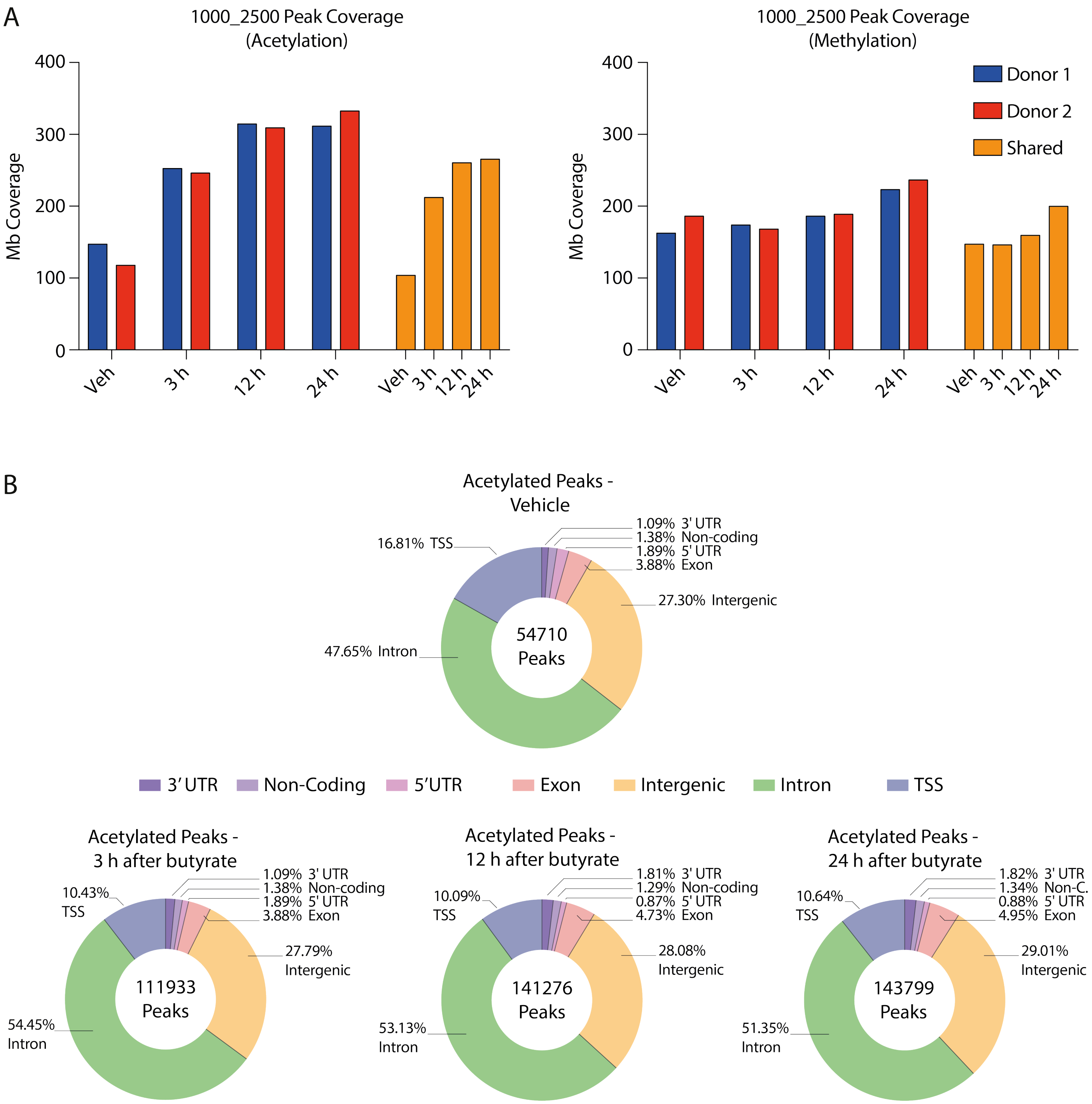


**Supplementary Figure 2. Butyrate treatment shifts the relative abundance of acetylation at genomic locations from TSS regions to intronic regions, without affecting histone methylation.** Chromatin immunoprecipitation (ChIP)-Seq specific for histone 3 lysine 27 acetylation (H3K27Ac) and histone 3 lysine 4 di-methylation (H3K4Me2) was performed after 3, 12 and 24 h of butyrate or 24 h vehicle ('0 h') treatment. (**A**) Megabase (Mb) coverage of histone acetylation (H3K27Ac, left) and methylation (H3K4Me2, right), using peak-calling parameter settings -size 1000 -minDist 2500. Donor 1 is indicated in blue and donor 2 is indicated in red. Overlap between the donors is indicated by the shared bars (yellow). (**B**) Genomic annotation of H3K27Ac peaks in vehicle treated human mast cells (upper donut graph) and redistribution of acetylation peaks induced by 3, 12 and 24 hours of butyrate treatment (lower donut graphs).

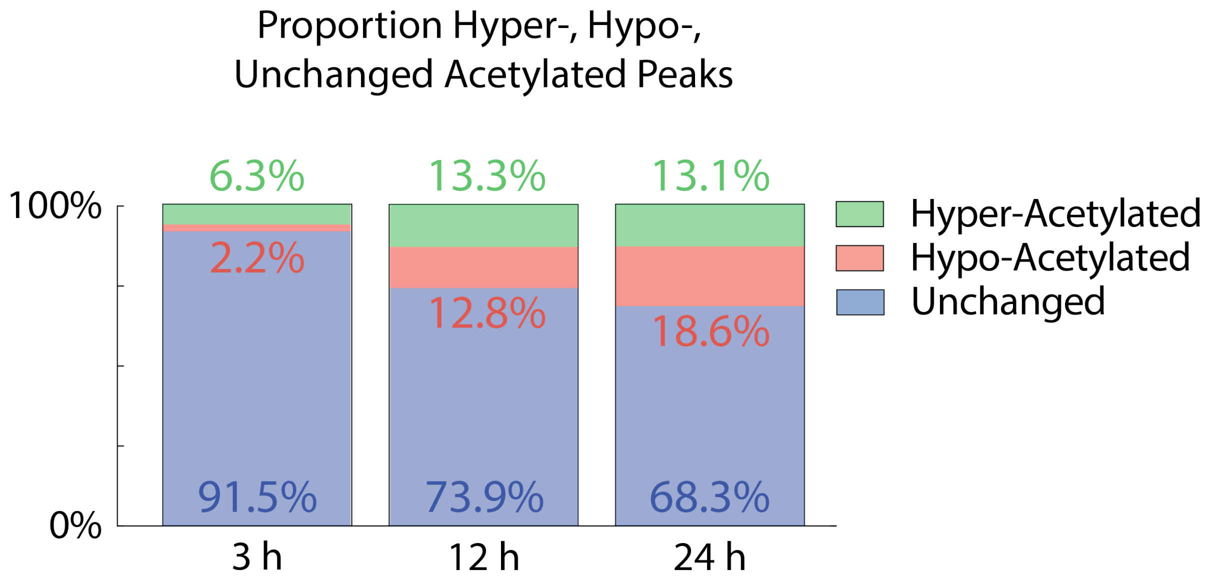


**Supplementary Figure 3. Most H3K27Ac+ regions at baseline are not significantly affected by butyrate treatment.** Differential enrichment analysis (fold change > 2 and adjusted P value < 0.05) was performed using DESeq2 to gain a quantitative picture of H3K27Ac dynamics upon butyrate treatment. Distribution of hyper-acetylated (in green), hypo-acetylated (in red) and unchanged (in purple) baseline (0 h) H3K27Ac+ peaks in response to 3, 12 and 24 hours of butyrate treatment. Data were obtained from human mast cells derived from two different donors.

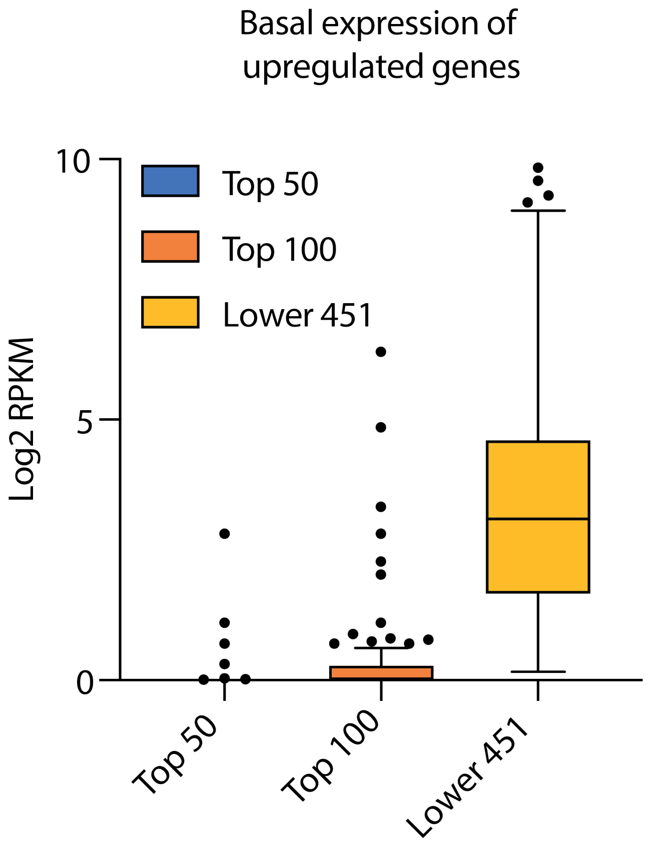


**Supplementary Figure 4. Strongly upregulated genes upon butyrate treatment display low levels of baseline acetylation.** Comparison of basal expression levels of upregulated genes, distinguishing between the top 50 (in blue), top 100 (in orange) and lower 451 (in yellow) upregulated genes.

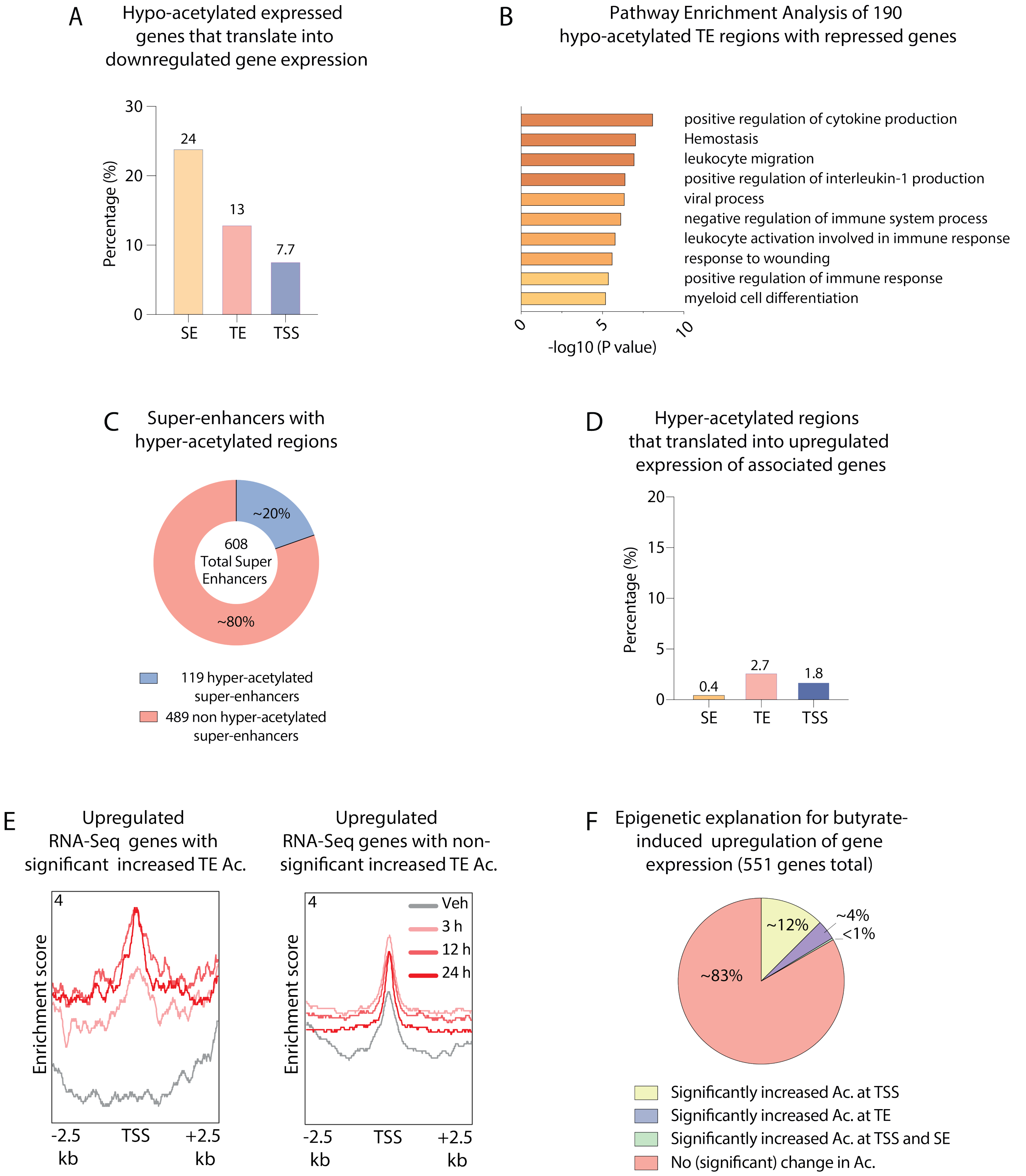


**Supplementary Figure 5. H3K27Ac acetylation at super-enhancers and associated transcriptional dynamics.** (**A**) The percentage of expressed hypo-acetylated genes that are downregulated upon butyrate treatment and associated with SE, TE or TSS. (**B**) Pathway enrichment analysis of 190 hypo-acetylated TE regions with repressed genes. (**C**) Proportion of identified SE that contain hyper-acetylated regions (in blue) and SE regions that do not intersect with a hyper-acetylated region (in light red). (**D**) The percentage of hyper-acetylated SE, TE or TSS regions linked to an upregulated gene. (**E**) Histograms of histone-acetylation at the TSS of upregulated genes with significant (left box) and non-significant (right box) hyper-acetylation at their TEs. The 0 h timepoint is indicated in light grey and TSS acetylation after butyrate treatment indicated in red. (**F**) Epigenetic explanation for butyrate-induced upregulation of gene expression. The proportion of upregulated genes with significant hyper-acetylated TSS (purple), TSS and SE (green), SE (orange), TE (yellow) and non-significant reduced acetylation (pink). Data were obtained from human mast cells derived from two different donors.

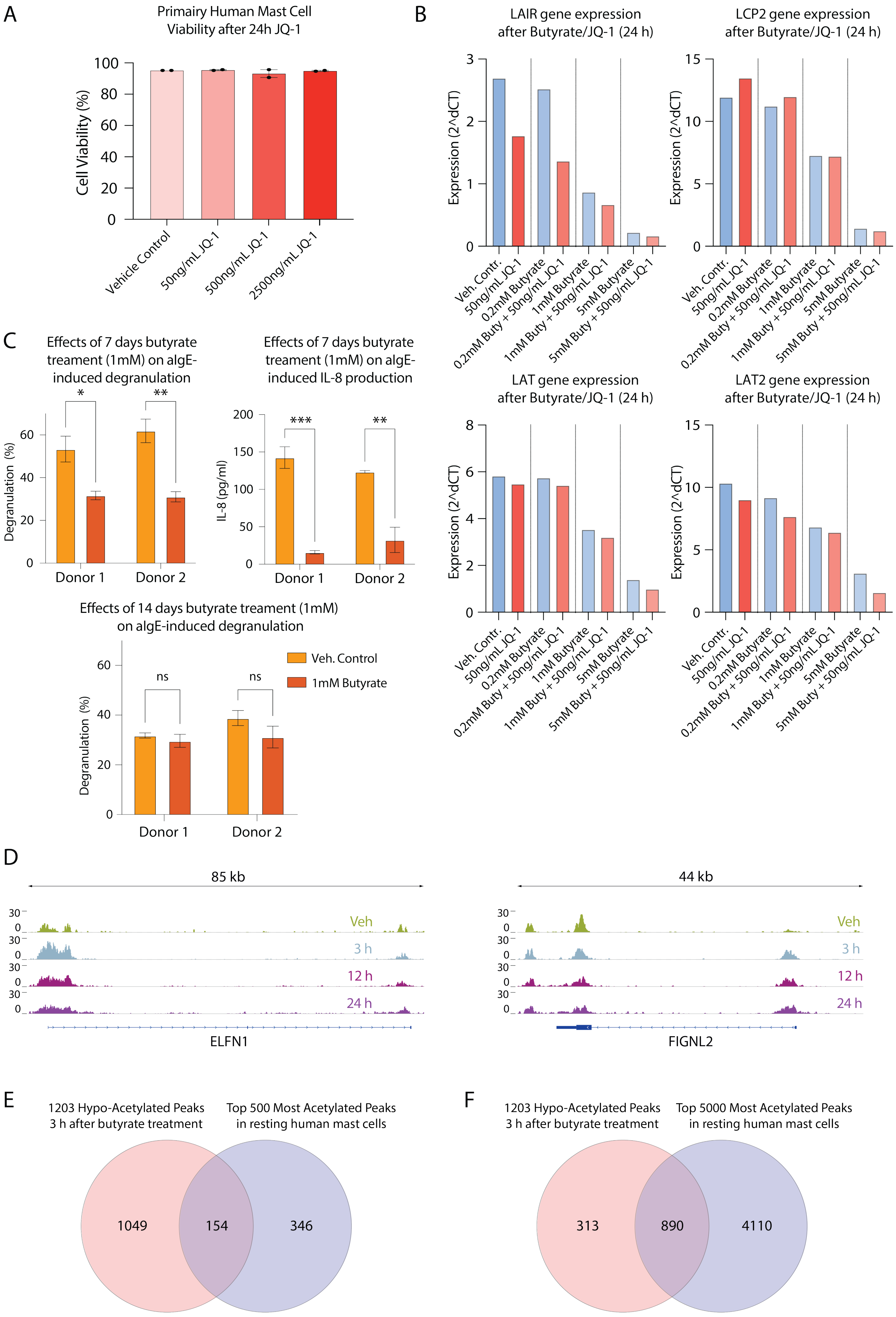
A
Primairy Human Mast Cell
Viability after 24h JQ-1
Cell Viability (%)
100
80
60
40
20
0
Vehicle Control
50ng/mL JQ-1
500ng/mL JQ-1
2500ng/mL JQ-1
B
LAIR gene expression
after Butyrate/JQ-1 (24 h)
LCP2 gene expression
after Butyrate/JQ-1 (24 h)
LAT gene expression
after Butyrate/JQ-1 (24 h)
LAT2 gene expression
after Butyrate/JQ-1 (24 h)
Expression (2^dCT)
Veh. Contr.
50ng/mL JQ-1
0.2mM Butyrate
0.2mM Buty + 50ng/mL JQ-1
1mM Butyrate
1mM Buty + 50ng/mL JQ-1
5mM Butyrate
5mM Buty + 50ng/mL JQ-1
C
Effects of 7 days butyrate treament (1mM) on aIgE-induced degranulation
Degranulation (%)
Effects of 7 days butyrate treament (1mM) on aIgE-induced IL-8 production
IL-8 (pg/ml)
*
**
***
Donor 1
Donor 2
Effects of 14 days butyrate treament (1mM) on aIgE-induced degranulation
ns
Veh. Control
1mM Butyrate
D
85 kb
44 kb
Veh
3 h
12 h
24 h
ELFN1
FIGNL2
E
1203 Hypo-Acetylated Peaks 3 h after butyrate treatment
Top 500 Most Acetylated Peaks in resting human mast cells
1049
154
346
F
1203 Hypo-Acetylated Peaks 3 h after butyrate treatment
Top 5000 Most Acetylated Peaks in resting human mast cells
313
890
4110

**Supplementary Figure 6. Extensively acetylated peaks are preferable targets of butyrate-induced hypo-acetylation, but in a selective manner.** (**A**) Percentage of viable mast cells after 24 hours treatment with JQ-1. (**B**) Gene expression (2^delta CT) of *LAIR*, *LCP2, LAT* and *LAT2* in human mast cells treated with varying concentrations of butyrate and/or JQ-1 for 24 h. (**C**) The effects of 7-14 days butyrate (1 mM) or vehicle treatment on human mast cell degranulation (as measured by the percentage of beta-hexosaminidase release) and IL-8 cytokine production. (**D**) Genomic browser view of representative examples of unaffected or (transient) hyper-acetylation at established acetylation peaks. (**E**) Venn diagram comparing the 1203 hypo-acetylated peaks after 3 hours of butyrate treatment with the top 500 most acetylated peaks in the mast cell epigenome. (**F**) Venn diagram comparing the 1203 hypo-acetylated peaks after 3 hours of butyrate treatment with the top 5000 most acetylated peaks in the mast cell genome. Data were obtained from human mast cells derived from three (one for panel B) different donors, with each condition represented by average values of 2-3 technical replicates. Data represent mean ± SEM (A) and ± SD (C), statistical significance was tested using a one-way ANOVA test.
